# Bacterial Lipoproteins Induce BAFF Production *via* TLR2/MyD88/JNK Signaling Pathways in Dendritic Cells

**DOI:** 10.3389/fimmu.2020.564699

**Published:** 2020-10-02

**Authors:** Jintaek Im, Jung Eun Baik, Dongwook Lee, Ok-Jin Park, Dong Hyun Park, Cheol-Heui Yun, Seung Hyun Han

**Affiliations:** ^1^Department of Oral Microbiology and Immunology, DRI, and BK21 Plus Program, School of Dentistry, Seoul National University, Seoul, South Korea; ^2^Department of Agricultural Biotechnology and Research Institute for Agriculture and Life Sciences, Seoul National University, Seoul, South Korea

**Keywords:** B-cell activating factor (BAFF), bacterial lipoproteins, Pam3CSK4, bone marrow–derived dendritic cells, TLR2/1, MyD88, JNK

## Abstract

B-cell activating factor (BAFF) plays a crucial role in survival, differentiation, and antibody secretion of B cells. Microbial products with B-cell mitogenic properties can indirectly promote expansion and activation of B cells by stimulating accessory cells, such as dendritic cells (DCs), to induce BAFF. Although bacterial lipoproteins are potent B-cell mitogen like lipopolysaccharides (LPSs), it is uncertain whether they can stimulate DCs to induce BAFF expression. Here, we evaluated the effect of bacterial lipoproteins on BAFF expression in mouse bone marrow–derived DCs. Lipoprotein-deficient *Staphylococcus aureus* mutant induced relatively low expression level of membrane-bound BAFF (mBAFF) and the mRNA compared with its wild-type strain, implying that bacterial lipoproteins can positively regulate BAFF induction. The synthetic lipopeptides Pam2CSK4 and Pam3CSK4, which mimic bacterial lipoproteins, dose-dependently induced BAFF expression, and their BAFF-inducing capacities were comparable to those of LPS in DCs. Induction of BAFF by the lipopeptide was higher than the induction by other microbe-associated molecular patterns, including peptidoglycan, flagellin, zymosan, lipoteichoic acid, and poly(I:C). Pam3CSK4 induced both mBAFF and soluble BAFF expression in a dose- and time-dependent manner. BAFF expression by Pam3CSK4 was completely absent in DCs from TLR2- or MyD88-deficient mice. Among various MAP kinase inhibitors, only JNK inhibitors blocked Pam3CSK4-induced BAFF mRNA expression, while inhibitors blocking ERK or p38 kinase had no such effect. Furthermore, Pam3CSK4 increased the DNA-binding activities of NF-κB and Sp1, but not that of C/EBP. Pam3CSK4-induced BAFF promoter activity *via* TLR2/1 was blocked by NF-κB or Sp1 inhibitor. Collectively, these results suggest that bacterial lipoproteins induce expression of BAFF through TLR2/MyD88/JNK signaling pathways leading to NF-κB and Sp1 activation in DCs, and BAFF derived from bacterial lipoprotein-stimulated DCs induces B-cell proliferation.

## Introduction

B cells are essential components for immune responses, including antibody production, antigen presentation, lymphoid organogenesis, cytokine expression, and T-cell differentiation (1). Regulation of B-cell expansion and activation is important to efficiently mediate host immunity. Multiple reports show that microbial products can be involved in expansion and activation of B cells directly and/or indirectly (2). For example, lipopolysaccharides (LPSs) of Gram-negative bacteria directly induce clonal expansion of B cells by interacting with Toll-like receptor 4 (TLR4)/MD-2 or RP105/MD-1 expressed on cellular membranes of B cells (3, 4). Additionally, LPS can also indirectly promote proliferation and activation of B cells by inducing B-cell activating factor (BAFF) production from antigen-presenting cells, including dendritic cells (DCs) and macrophages (5–7).

BAFF, which belongs to the tumor necrosis factor (TNF) superfamily, plays a fundamental role in the maturation, proliferation, and survival of B cells, as well as immunoglobulin class switching and production (8, 9). Although various cell types can produce BAFF, including activated T-lymphocytes, neutrophils, and epithelial cells (10–12), BAFF is produced predominantly by antigen-presenting cells, including DCs, monocytes, and macrophages, in response to cytokines such as interferon (IFN)-*γ*, transforming growth factor (TGF)-β, and microbial products such as LPS (6, 7, 10). Upon activation of cells, BAFF is initially produced in a membrane-bound form and then secreted in a soluble form after cleavage by furin-like convertase (13). Subsequently, soluble BAFF (sBAFF) binds to three distinct receptors on B cells: BAFF-receptor (BAFF-R), transmembrane activator and calcium modulator and cyclophilin ligand interactor (TACI), and B-cell maturation antigen (BCMA) (14). As these BAFF receptors have different biological functions in B cells, their distinct expression patterns during B-cell development determine the roles of BAFF at each developmental stage (11). For example, because BAFF-R is expressed on immature B cells rather than mature B cells, BAFF binding to BAFF-R mediates maturation and survival in immature B cells. However, because TACI is predominantly expressed on mature B cells, such as plasma cells, but not on immature B cells, BAFF binding to TACI promotes class-switch recombination and survival of plasma cells (13, 15).

In addition to the role of BAFF in B-cell activation, it is also involved in initiation and progression of inflammatory responses by promoting T helper 1 (Th1) and Th17 pathways (16). According to previous studies, BAFF stimulated T cell polarization toward a Th1 phenotype resulting in increased Th1 cytokine production. BAFF also promoted Th17 responses directly by the enhancing differentiation of naive T cells into Th17 cells (17), and indirectly by promoting release of pro-Th17 cytokines, such as interleukin (IL)-23, IL-1β, and TGF-β from monocytes (18). Furthermore, clinical studies showed that BAFF levels in serum have a positive correlation with the initiation as well as severity of several autoimmune and inflammatory diseases characterized by chronic inflammation, such as inflammatory bowel disease, periodontitis, and systemic sclerosis (16, 19, 20).

Bacterial lipoproteins are abundant cell-wall components involved in maintenance of cell division, antibiotic resistance, and adhesion to host tissues (21). Bacterial lipoproteins are also considered as major microbial products with a strong ability to induce proinflammatory cytokines, such as TNF-α, IL-6, IL-8, and IL-12 (22, 23). Despite some exceptions, Gram-positive bacterial cell walls contain primarily diacylated lipoproteins, while Gram-negative bacteria have triacylated lipoproteins (24). Triacylated and diacylated lipoproteins are initially sensed by TLR2/1 and 2/6 heterodimers, respectively, on host cells (25). The recognition then induces recruitment of the downstream intracellular signal molecule MyD88, promoting phosphorylation of mitogen-activated protein (MAP) kinases and activation of NF-κB, which are essential for gene expression of various proinflammatory molecules (26).

Although previous studies have reported that bacterial lipoproteins promote expansion and activation of B cells by interacting directly with B cells (27, 28), the indirect B-cell activating potency through BAFF induction is poorly understood. The ability of bacterial lipoproteins to induce expression of proinflammatory cytokines suggests that bacterial lipoproteins can indirectly promote proliferation and activation of B cells by inducing BAFF expression. In the present study, we evaluated whether bacterial lipoproteins can induce BAFF expression in mouse bone marrow–derived DCs using a lipoprotein-deficient *Staphylococcus aureus* mutant strain and synthetic lipopeptides mimicking bacterial lipoproteins, and investigated the underlying intracellular mechanisms.

## Materials and Methods

### Reagents and Chemicals

Pam3CSK4, Pam2CSK4, poly(I:C), flagellin, zymosan, and peptidoglycan (PGN) were purchased from InvivoGen (San Diego, CA, USA). Lipoteichoic acid (LTA) from *S. aureus* was prepared as previously described (29). Standard LPS (stdLPS) from *Escherichia coli* O111:B4 prepared by classical methods using phenol-water mixture extraction, and ultra-pure LPS (upLPS) prepared by successive enzymatic hydrolysis and phenol-triethylamine-deoxycholate extraction to remove other bacterial components from stdLPS were also obtained from InvivoGen. Fetal bovine serum (FBS), Roswell Park Memorial Institute (RPMI) medium and Dulbecco’s modified Eagle medium (DMEM) were obtained from HyClone (Logan, UT, USA). MAP kinase inhibitors, including PD98059 for ERK, SB202190 for p38, and SP600125 and JNK V inhibitor for JNK, and transcription factor inhibitors, such as BAY11-7082 for NF-κB, and mithramycin A for Sp1, were obtained from Sigma-Aldrich (St. Louis, MO, USA). All other reagents and chemicals were from Sigma-Aldrich unless stated otherwise.

### Preparation of Bone Marrow–Derived DCs

C57BL/6 mice were purchased from OrientBio (Gyeonggi-do, Korea) and TLR2- or MyD88-deficient mice with C57BL/6 background were kindly provided by Prof. Shizuo Akira (Osaka University, Osaka, Japan). DCs were prepared as previously described (30). Briefly, bone marrow cells were obtained by flushing the marrow space of tibiae and femurs with phosphate-buffered saline (PBS). After removing red blood cells (RBCs) using an RBC-lysing buffer (Sigma-Aldrich), bone marrow cells were cultured with RPMI-1640 medium supplemented with 10% heat-inactivated FBS, 100 U/ml of penicillin, and 100 µg/ml of streptomycin in the presence of 20 ng/ml of mouse granulocyte/macrophage–colony stimulating factor (Peprotech, Rocky Hill, NJ, USA) and 50 µM of 2-mercaptoethanol (Sigma-Aldrich) for 6 days at 37°C. On day 6 post-culture, DCs were collected by centrifugation and used for experiments.

### Preparation of Ethanol-Killed *S. aureus*

Wild-type *S. aureus* parental strain RN4220 and its lipoprotein-deficient mutant (*Δlgt*) were provided by Prof. Bok Luel Lee (Pusan National University, Pusan, Korea) (31). Wild-type strain was grown in Luria–Bertani (LB) broth (BD Biosciences, Franklin, NJ, USA) at 37°C and *Δlgt* strain was grown in LB broth containing 10 µg/ml erythromycin at 37°C with constant shaking. After the harvest by centrifugation at 8,000 × *g* for 15 min, the bacteria were killed by immersion in 70% ethanol for 90 min with constant shaking and then washed with PBS. No colonies were observed (data not shown) when the ethanol-killed bacteria were plated onto an LB agar plate containing 1.5% agar. Bacterial pellet, wet weight-based, per colony forming unit (CFU) was measured by spotting assay and each fifty microgram of strain was approximately equal with 2.5 × 10^7^ CFU (31, 32).

### Flow Cytometry

DCs were treated with 50 µg/ml of the ethanol-killed *S. aureus* strain for 48 h, or with Pam3CSK4 at 0–10 μg/ml for 0 to 72 h. Following stimulation, the cells were stained with anti-mouse antibodies conjugated with fluorescent dyes as previously described (33). For membrane-bound BAFF (mBAFF) analysis, DCs were stained with anti-mouse BAFF antibodies conjugated with fluorescein isothiocyanate (FITC) (Enzo Life Science, Plymouth Meeting, PA, USA) together with anti-mouse CD11c antibodies conjugated with allophycocyanin (APC) (BD Biosciences). Additionally, DCs were stained with antibodies for mouse TLR1 conjugated with Alexa Fluor 647 or TLR2 conjugated with phycoerythrin (PE) (e-Bioscience, San Diego, CA, USA). The cells were then subjected to flow cytometry (FACSCalibur) with CellQuest software (BD Biosciences). Data were analyzed using FlowJo software (Tree Star, San Carlos, CA, USA).

### Enzyme-Linked Immunosorbent Assay (ELISA)

DCs were stimulated with Pam3CSK4 at 0, 0.1, 1.0, or 10 μg/ml for 0, 3, 6, 12, 24, 48, or 72 h. After stimulation, culture supernatants were collected and sBAFF levels were measured using a commercially-available mouse-BAFF ELISA kit (R&D Systems, Minneapolis, MN, USA) according to the manufacturer’s instructions.

### Reverse Transcription-Polymerase Chain Reaction (RT-PCR)

DCs were treated with 50 μg/ml of ethanol-killed *S. aureus* strains for 48 h, or with Pam3CSK4 at 0, 0.1, 1.0, or 10 μg/ml for 0, 3, 6, 9, 12, 24, 48, or 72 h. For experiments using MAP kinase inhibitors, cells were pre-treated with 0, 2.5, 5, 10 or 20 μM of each MAP kinase inhibitor for 1 h followed by stimulation with 10 μg/ml of Pam3CSK4 for an additional 72 h. Dimethyl sulfoxide (DMSO; 0.1%) was used as a vehicle control for each MAP kinase inhibitor. To measure TLRs mRNA expression levels, the cells were stimulated with 10 μg/ml of Pam3CSK4 for 48 h. After stimulation, total RNA was isolated using a TRIzol reagent (Invitrogen, Carlsbad, CA, USA) and cDNA was synthesized as previously described (34). Amplification of cDNA by PCR was conducted using 0.5 unit of rTaq DNA polymerase and 10 picomole of primers specific to mouse BAFF or TLR1 to TLR9 (**Table 1**). PCR products were separated on a 1.5% agarose gel containing ethidium bromide and visualized using a gel image analyzer (Syngene InGenius3, Synoptics, Cambridge, UK). Expression of each target gene was presented as percentage change by densitometric analysis (GeneTools analysis, Synoptics).

**Table 1:** Sequences of primers used for RT-PCR analysis.

Target gene	Primer orientation	Primer sequence (5’-3’)
BAFF	Forward Reverse	TCAGAATATGCCCAAAACACTG AATCTCCCTGCATTCTGAACAT
TLR1	Forward Reverse	ACTCAGGCGAGCAGAGGCAA GGCTGACTGTTGGGTGGCACA
TLR2	Forward Reverse	GACTCACAGCAGCCATGAAA TCGCGGATCGACTTTAGACT
TLR3	Forward Reverse	TGGGAACGGGGGTCCAACTG AGAGCGAGGGGACAGACGCT
TLR4	Forward Reverse	GCCCCGCTTTCACCTCTGCC AGCCCCAGGTGAGCTGTAGCA
TLR5	Forward Reverse	CTGCTTTCCCGAGCCCAGCG GGTGCGTGGGGGAACTCAGC
TLR6	Forward Reverse	GCTGCCCTATGGCGAGCCTG CCACCGGTCGGGGCCAAAAG
TLR7	Forward Reverse	AGGCTCTGCGAGTCTCGGTT TGCAGTCCACGATCACATGG
TLR8	Forward Reverse	TGGCTGCTCTGGTTCACCACC GGGCCACTGGAGGATGGAGC
TLR9	Forward Reverse	ACGCAGCGCCCAAACTCTCC GCGGTCTTCCAACAGGCGCT
β-actin	Forward Reverse	GTGGGGCGCCCCAGGCACCA CTCCTTAATGTCACGCACGAT

### Transfection and Luciferase Reporter Gene Assay

HEK-293 cells stably transfected with mouse TLR1- and TLR2-expression plasmids (293/TLR1-TLR2) and the control plasmid (293/Null) were obtained from InvivoGen, and cultured in DMEM medium containing 10% heat-inactivated FBS, 100 U/ml of penicillin, and 100 µg/ml of streptomycin in the presence of 10 µg/ml of blasticidin S (InvivoGen) at 37°C. The mouse BAFF promoter luciferase reporter construct was provided by Prof. Eun-Yi Moon at Sejong University, Korea (35). Transfection followed by reporter gene assay was conducted as previously described (36). Briefly, 293/TLR1-TLR2 or 293/Null cells were transiently transfected with the mouse BAFF promoter luciferase reporter construct together with *Renilla* luciferase construct (pRL-TK; Promega, Madison, WI, USA) using a TransFectin lipid reagent (Bio-Rad, Hercules, CA, USA). The cells were then stimulated with 0, 0.1, 1.0, or 10 μg/ml of Pam3CSK4 for 24 h. For experiments using transcription factor-specific inhibitors, the cells were pre-treated with various concentrations of BAY11-7082 or mithramycin A for 1 h followed by stimulation with 10 μg/ml of Pam3CSK4 for an additional 24 h. DMSO (0.1%) was used as a vehicle control for each transcription factor inhibitor. After the stimulation, the cells were lysed with a luciferase assay buffer (Promega) and luciferase activity was measured using a luminometer (Victor 3, PerkinElmer, Waltham, MA, USA). Data were expressed as relative luciferase activity after normalization with pRL-TK in the same cytoplasmic extract to correct for differences in transfection efficiency.

### Electrophoretic Mobility Shift Assay

Electrophoretic mobility shift assay was conducted as previously described (37). Briefly, DCs were stimulated with 10 μg/ml of Pam3CSK4 for 0–8 h and nuclear extracts were prepared. The nuclear extracts were incubated with double-stranded deoxyoligonucleotide probes containing the consensus recognition sites of NF-κB, Sp1, or C/EBP end-labeled with [*γ*-^32^P]-dATP. After incubation, reactants were subjected to electrophoresis using 4.8% polyacrylamide gels. After drying, gels were subjected to autoradiography. To confirm specific binding, 1 picomole of an unlabeled probe was used as a cold competitor.

### B-Cell Proliferation Assay

B-cell proliferation in response to the conditioned media of DCs stimulated with Pam3CSK4 was conducted as previously described (27). To prepare conditioned media, DCs were stimulated with 10 μg/ml of Pam3CSK4 for 12 h, washed with PBS twice, and further incubated for an additional 60 h. Then, the conditioned media were collected by centrifugation at 16,400 × *g* for 5 min. Splenocytes were isolated from C57BL/6 mice by homogenizing the spleens and removing erythrocytes using RBC lysing buffer (Sigma-Aldrich) and labeled with 10 μM carboxyfluorescein succinimidyl ester (CFSE) in PBS at 37°C for 20 min. The CFSE-labeled splenocytes were seeded on 96-well U-bottom plate and stimulated with the indicated amount of conditioned media, 10 µg/ml of Pam3CSK4, or 1 µg/ml of LPS for 72 h. Then, the cells were stained with antibody for mouse CD45R/B220 conjugated with PerCP and B-cell proliferation was determined by monitoring CFSE levels of the B220-positive cell population using a flow cytometry (FACSCalibur) with CellQuest software (BD Biosciences). Data were analyzed using FlowJo software (Tree Star).

### Statistical Analysis

All experiments were performed at least three times and the mean values ± standard deviation were determined from triplicate samples for each treatment group. Statistical significance was examined with Student’s *t*-test.

## Results

### Lipoprotein-Deficient *S. aureus* Induces Lower BAFF Expression Than Does the Wild-Type Strain in DCs

**Figure 1:**
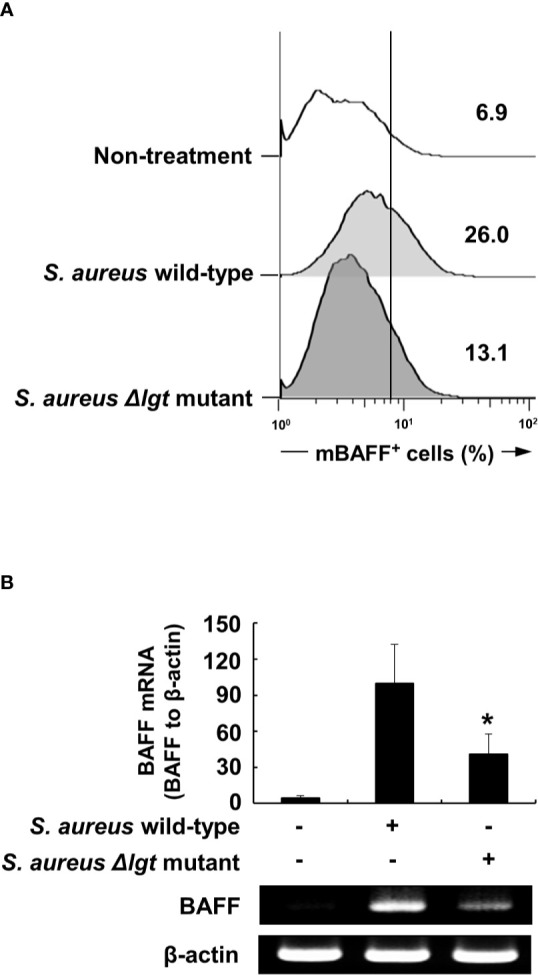
Lipoprotein-deficient *Staphylococcus aureus* strain (Δ*lgt*) induces lower BAFF expression compared with the wild-type strain in DCs. **(A)** DCs were treated with 50 µg/ml of ethanol-killed *S. aureus* wild-type or lipoprotein-deficient strain (*Δlgt*) for 48 h, stained with antibodies for mouse CD11c conjugated with APC and BAFF conjugated with FITC, and subjected to flow cytometry. The histograms are representative results of flow cytometry for membrane-bound BAFF (mBAFF) from each treatment group, and the value in each histogram is the percentage of mBAFF-positive cells among 10,000 events of CD11c-positive cells. **(B)** DCs were treated with 50 µg/ml of ethanol-killed *S. aureus* wild-type or *Δlgt* strains for 48 h. BAFF mRNA expression was then determined by RT-PCR. *Upper*, BAFF mRNA expression normalized with that of β-actin is presented as percentage change ± standard deviation of three separate experiments against the wild-type treatment group, set as 100% by densitometric analysis. *Lower*, a representative BAFF mRNA expression determined by RT-PCR. Asterisk (*) indicates statistical significance at *p* < 0.05 between the *S. aureus* wild-type and Δ*lgt* strain treatment groups.

We initially examined whether bacterial lipoproteins are involved in BAFF production of DCs using a mutant strain of lipoprotein-deficient *S. aureus* (*Δlgt*). Because prolipoprotein diacylglycerol transferase (Lgt) is known to mediate the transfer of diacylglycerol to the sulfhydryl moiety of a cysteine residue in the lipobox of pre-prolipoproteins, which leads to maturation of bacterial lipoprotein, the *Δlgt* strain exhibited no evidence of lipoprotein synthesis (24). Although Gram-positive and Gram-negative bacteria are known to contain diacylated and triacylated lipoproteins, respectively, *S. aureus*, a major Gram-positive bacterial pathogen, uniquely expresses both diacylated and triacylated lipoproteins on its cell walls (38). The *S. aureus Δlgt* strain can therefore be used as an appropriate model to examine the effects of two different forms of bacterial lipoproteins on BAFF production. When DCs were treated with ethanol-killed wild-type *S. aureus* or *Δlgt* strains for 48 h, the *Δlgt* strain displayed relatively reduced mBAFF induction compared with that in the wild-type strain (**Figure 1A**). In accordance with results of flow cytometry, expression of BAFF mRNA induced by the *Δlgt* strain was lower than that of the wild-type strain (**Figure 1B**). These results suggest that bacterial lipoproteins can induce BAFF expression in DCs.

### Synthetic Lipopeptide, Pam3CSK4, Induces Relatively Higher Levels of BAFF Expression than Other Microbial Products in DCs

**Figure 2:**
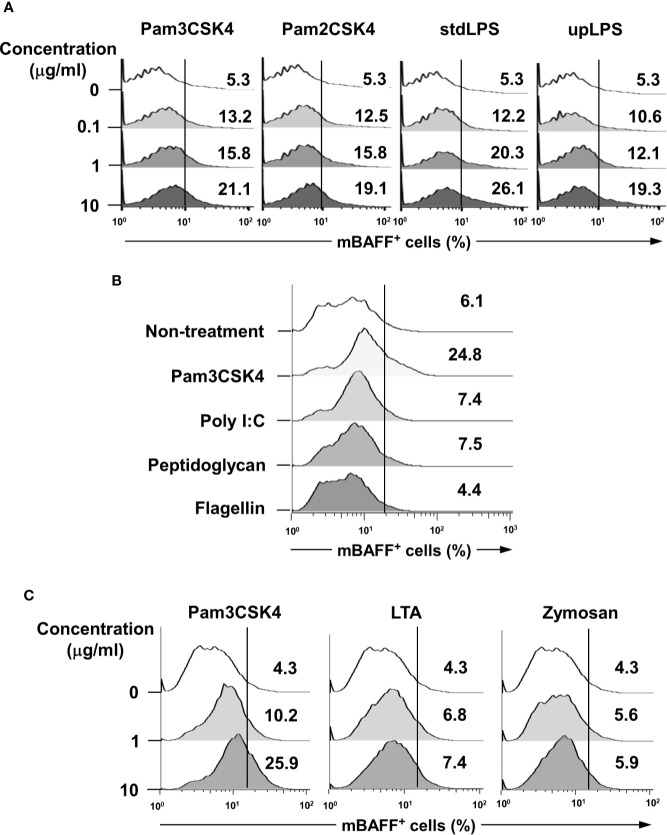
Lipopeptides mimicking bacterial lipoproteins induce higher BAFF expression compared with other microbial products in DCs. **(A)** DCs were stimulated with 0-10 μg/ml of Pam3CSK4, Pam2CSK4, standard LPS (stdLPS), or ultra-pure LPS (upLPS) for 48 h. **(B)** DCs were stimulated with Pam3CSK4 (10 μg/ml), poly(I:C) (25 μg/ml), peptidoglycan (10 μg/ml), or flagellin (100 ng/ml) for 48 h. **(C)** DCs were stimulated with 0–10 μg/ml of Pam3CSK4, LTA from *S. aureus*, or zymosan for 48 h. After stimulation, the cells were stained with CD11c antibodies conjugated with APC and BAFF antibodies conjugated with FITC, and subjected to flow cytometry. The histograms are representative results from flow cytometry for mBAFF from each treatment group, and the value given in each histogram is the percentage of mBAFF-positive cells among 10,000 events of CD11c-positive cells.

To validate whether bacterial lipoproteins can induce BAFF expression, we evaluated the effects of Pam3CSK4 and Pam2CSK4 (the respective synthetic triacylated and diacylated lipopeptides that mimic bacterial lipoproteins) (39, 40), on mBAFF expression in DCs. As shown in **Figure 2A**, both Pam3CSK4 and Pam2CSK4 enhanced mBAFF expression in a dose-dependent fashion. In addition, since it has been reported that some commercially-available LPS preparations are contaminated with other bacterial components, such as lipoproteins and PGN (41), we also confirmed the capacity of upLPS that was prepared by enzymatic removal of other bacterial components from stdLPS to induce mBAFF expression on DCs. Like stdLPS, upLPS also induced mBAFF expression in a dose-dependent manner (**Figure 2A**). Next, we compared the BAFF-inducing ability of lipopeptides using Pam3CSK4 with that of other microbial products, including poly(I:C), flagellin, and PGN, and found that only Pam3CSK4 induced mBAFF production in DCs (**Figure 2B**). As both Pam2CSK4 and Pam3CSK4 commonly utilize TLR2 for the recognition, BAFF-inducing abilities of other TLR2 ligands were also tested. The mBAFF was dose-dependently induced by Pam3CSK4, but not by LTA or zymosan (**Figure 2C**). These results demonstrate that bacterial lipoproteins have a potent BAFF-inducing ability than other microbial products.

### Pam3CSK4 Induces Expression of BAFF at Both Protein and mRNA Levels

**Figure 3:**
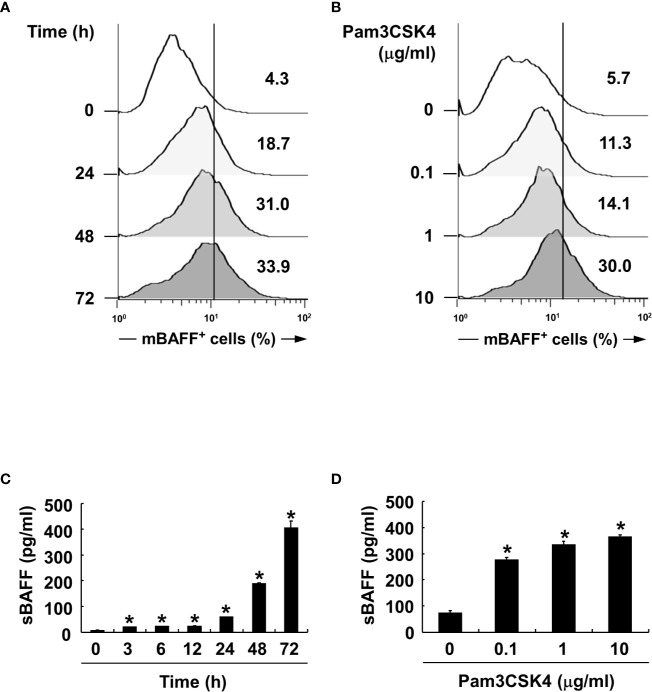
Pam3CSK4 increases BAFF expression in a dose- and time-dependent manner. **(A, C)** For time experiments, the DCs were stimulated with 10 μg/ml of Pam3CSK4 for 0–72 h. **(B, D)** For dose experiments, the cells were stimulated with 0-10 μg/ml of Pam3CSK4 for 72 h. **(A, B)** After stimulation, the cells were stained with antibodies for mouse CD11c conjugated with APC and BAFF conjugated with FITC, and subjected to flow cytometry. The histograms are representative results from flow cytometry for mBAFF, and the value given in each histogram is the percentage of mBAFF-positive cells among 10,000 events of CD11c-positive cells. **(C, D)** At the end the stimulation, culture media were collected and soluble BAFF (sBAFF) levels were measured by ELISA. Values are the mean ± standard deviation of three separate experiments. Asterisk (*) indicates statistical significance at *p* < 0.05 between the non-treatment and treatment groups.

**Figure 4:**
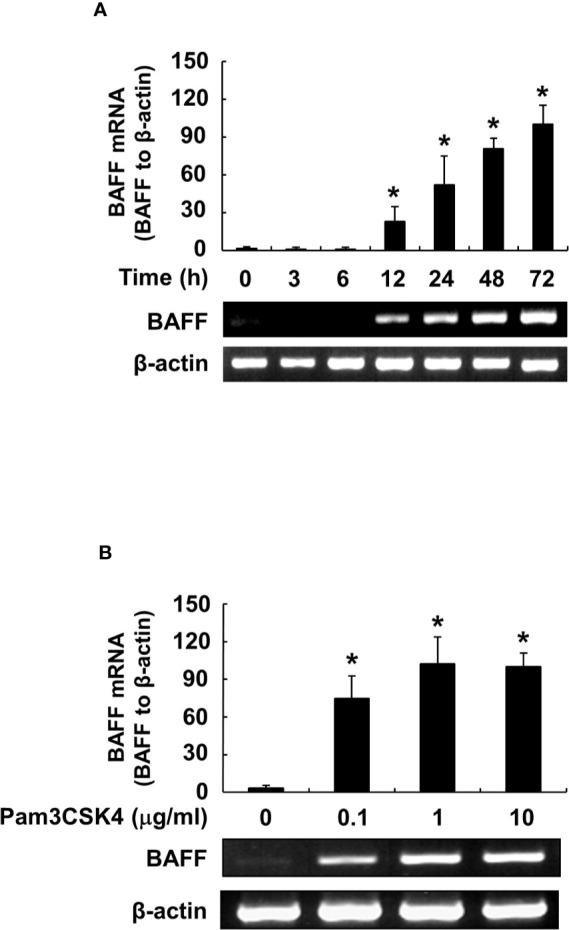
Pam3CSK4 induces an increase in BAFF mRNA levels. **(A)** DCs were stimulated with 10 μg/ml of Pam3CSK4 for 0–72 h, or **(B)** with Pam3CSK4 at 0-10 μg/ml for 72 h. Total RNA was isolated and cDNA was synthesized as described in the Materials and Methods. The cDNA was subjected to RT-PCR to determine expression levels of BAFF mRNA. *Upper*, β-actin–normalized BAFF mRNA expression is presented as the percentage change ± standard deviation of three separate experiments against the group showing the highest ratio [72 h and 10 μg/ml treatment group in **(A)** and **(B)**, respectively], set as 100% by densitometric analysis. *Lower*, a representative BAFF mRNA expression determined by RT-PCR. Asterisk (*) indicates statistical significance at *p* < 0.05 between the non-treatment and treatment groups.

We also confirmed BAFF induction by Pam3CSK4 in both mRNA and protein expression levels at various time points and concentrations of Pam3CSK4. When DCs were stimulated with Pam3CSK4 at 10 µg/ml for 0–72 h, or with 0–10 µg/ml of Pam3CSK4 for 72 h, mBAFF expression gradually increased in time-dependent (**Figure 3A**) and dose-dependent (**Figure 3B**) manners. Because mBAFF can be secreted in a soluble form after cleavage by furin-like convertases (13), we also measured sBAFF levels under the conditions used for the mBAFF experiment. As shown in **Figures 3C**, **D**, sBAFF levels were also enhanced by Pam3CSK4 in time- and dose-dependent fashions. As production of both mBAFF and sBAFF peaked at 10 µg/ml of Pam3CSK4 and 72 h after Pam3CSK4 stimulation, these conditions were chosen for the following experiments. Next, steady-state levels of BAFF mRNA were measured in DCs exposed to various concentrations of Pam3CSK4 for different time periods. In DCs treated with 10 µg/ml of Pam3CSK4 for 0–72 h, BAFF mRNA expression gradually increased with the extension of time (**Figure 4A**). When DCs were treated with 0–10 µg/ml of Pam3CSK4 for 72 h, BAFF mRNA expression increased to a dose of 1 µg/ml, and nearly plateaued at 10 µg/ml of Pam3CSK4 (**Figure 4B**). For subsequent BAFF mRNA analysis experiments, 10 µg/ml of Pam3CSK4 for 72 h was selected because the expression of BAFF mRNA by Pam3CSK4 peaked under this condition.

### TLR2 and MyD88 Are Necessary for Pam3CSK4-Induced BAFF Production

**Figure 5:**
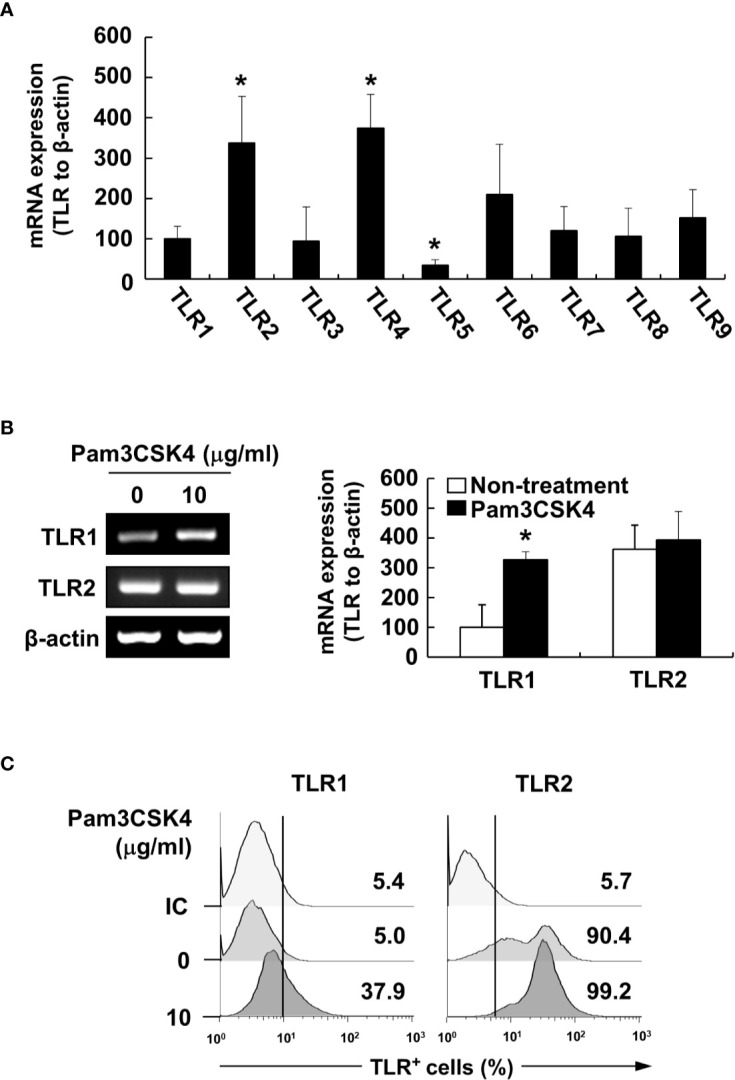
Pam3CSK4 enhances TLR1 expression. **(A)** Total RNA was isolated from DCs and subjected to RT-PCR to determine TLR1 to TLR9 mRNA expression levels. Each TLR mRNA expression normalized with that of β-actin is presented as the percentage change ± standard deviation of three separate experiments against TLR1 mRNA expression, set as 100% by densitometric analysis. Asterisk (*) indicates statistical significance at *p* < 0.05 between the TLR1 and other TLRs groups. **(B)** DCs were stimulated with 10 μg/ml of Pam3CSK4 for 48 h and isolated total RNA was subjected to RT-PCR to determine TLR1 or TLR2 mRNA expression levels. *Left*, a representative TLR1 and TLR2 mRNA expression determined by RT-PCR. *Right*, TLR1 and TLR2 mRNA expression normalized with that of β-actin is presented as the percentage change ± standard deviation of three separate experiments against non-treated TLR1 mRNA expression, set as 100% by densitometric analysis. Asterisk (*) indicates statistical significance at *p* < 0.05 between the non-treatment and treatment groups. **(C)** After stimulation with Pam3CSK4 at 10 μg/ml for 48 h, DCs were stained with antibodies for TLR1 conjugated with Alexa Fluor 647 or TLR2 conjugated with PE, and then subjected to flow cytometry. Mouse IgG2a conjugated with Alexa Fluor 647 were used as an isotype control (IC) antibody. The histograms are representative results from flow cytometry for TLR1 or TLR2 from each treatment group. The value given in each histogram is the percentage of TLR1- or TLR2-positive cells among 10,000 events of CD11c-positive cells.

**Figure 6:**
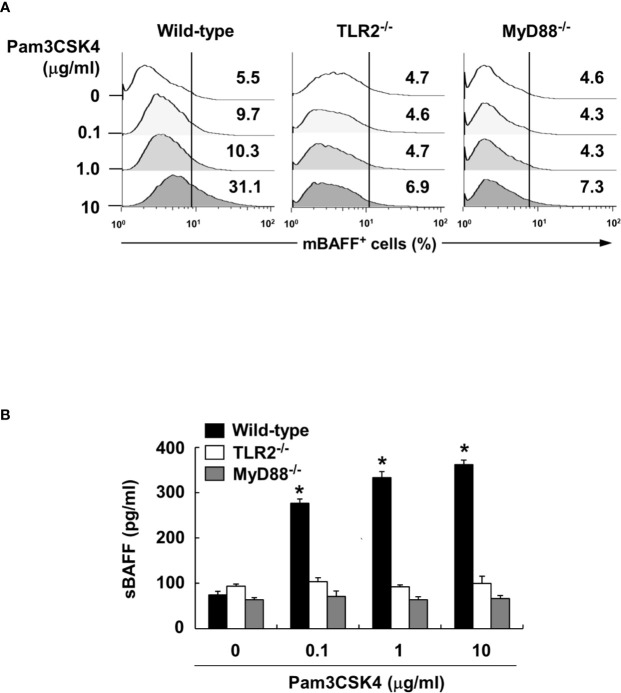
Pam3CSK4-induced BAFF expression is mediated through TLR2 and MyD88. **(A)** DCs from wild-type, TLR2- or MyD88-deficient mice were stimulated with Pam3CSK4 at 0-10 μg/ml for 72 h. The cells were then stained with antibodies for mouse CD11c conjugated with APC and BAFF conjugated with FITC, and subjected to flow cytometry. The histograms are representative results from flow cytometry for mBAFF, and the value in each histogram is the percentage of mBAFF-positive cells among 10,000 events of CD11c-positive cells. **(B)** Under the same culture condition, sBAFF levels in culture media were measured by ELISA. The values are the mean ± standard deviation of three separate experiments. Asterisk (*) indicates a significant difference at *p* < 0.05 between the non-treatment and treatment groups.

To characterize the underlying intracellular mechanisms that explain why bacterial lipoproteins have a relatively greater BAFF-inducing ability compared with those of other microbial products, we initially examined the relative mRNA expression levels of their cellular receptors, TLRs, on DCs. According to previous reports, poly(I:C), LPS, and flagellin are sensed by TLR3, TLR4, and TLR5, respectively, while Pam2CSK4 and Pam3CSK4 are recognized by TLR2/6 and TLR2/1 heterodimers, respectively (25). Whether LTA is sensed by TLR2 homodimers (42) or TLR2/1 or TLR2/6 heterodimers (43), TLR2 is necessary for cellular recognition of LTA. As shown in **Figure 5A**, TLR2, TLR4, and TLR6 mRNA levels were relatively high, while TLR1, TLR3, and TLR5 mRNA expressions were lower than those of the other TLRs. These results imply that the TLR expression on DCs reflected differences in the BAFF-inducing ability of the tested microbial products, except for TLR2 ligands such as LTA and zymosan. However, while the TLR2 mRNA level was higher than that of other TLRs, lower TLR1 mRNA expression makes it difficult to explain the high BAFF-inducing ability of Pam3CSK4, which is sensed by TLR2/1 heterodimer. Because a previous report utilizing leukocytes showed that TLR expression can be changed by stimulation of bacterial components (44), we examined whether low TLR1 expression can be altered by Pam3CSK4 at both the protein and mRNA levels. When DCs were stimulated with 10 μg/ml of Pam3CSK4, TLR1, but not TLR2, expression was dramatically enhanced by Pam3CSK4 stimulation at both the protein and mRNA level (**Figures 5B**, **C**), suggesting that enhanced TLR1 expression by Pam3CSK4 is likely to be involved in recognition of Pam3CSK4 with TLR2. After recognition of Pam3CSK4 *via* TLR2 and TLR1, the subsequent intracellular signaling cascades are mediated by MyD88 and its adaptor molecules (45). We therefore examined the role of MyD88 in Pam3CSK4-induced BAFF expression using DCs from MyD88-deficient mice (MyD88^−/−^) and TLR2-deficient mice (TLR2^−/−^). In accordance with BAFF expression by Pam3CSK4 in DCs from TLR2^−/−^ mice, BAFF production by Pam3CSK4 in DCs from MyD88^−/−^ mice was nearly eliminated for both mBAFF and sBAFF compared with such activity in wild-type mice (**Figures 6A, B**). These results suggest that Pam3CSK4-induced BAFF expression in DCs is mediated by a TLR2/MyD88-dependent pathway.

### JNK MAP Kinase, NF-κB, and Sp1 Are Involved in Transcriptional Regulation of the Pam3CSK4-Induced BAFF Expression in DCs

**Figure 7:**
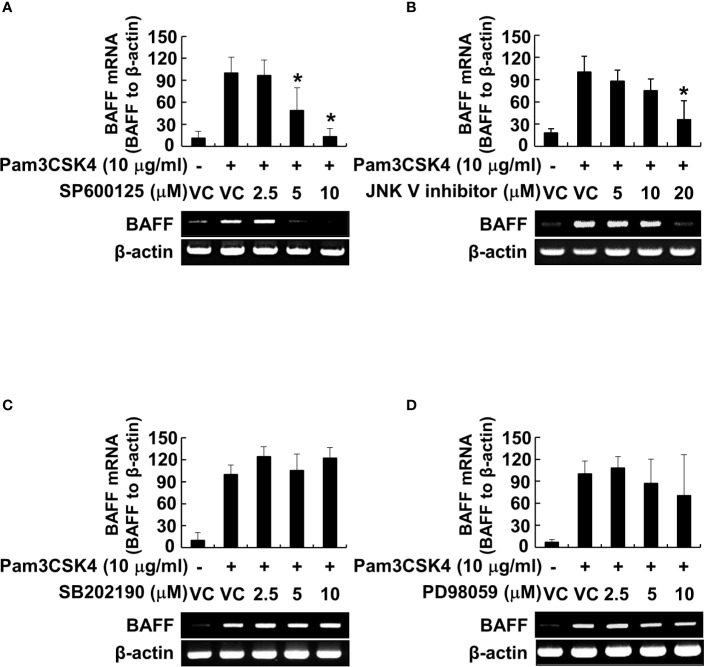
JNK MAP kinase is essential for Pam3CSK4-induced BAFF expression at the transcriptional level. **(A–D)** DCs were pre-treated with 0–20 μM of the indicated MAP kinase inhibitor (SP600125 and JNK V inhibitor for JNK inhibition, SB202190 for p38 inhibition, or PD98059 for ERK inhibition) for 1 h followed by stimulation with 10 μg/ml of Pam3CSK4 for an additional 72 h. Dimethyl sulfoxide (DMSO; 0.1%) was used as a vehicle control (VC) for each MAP kinase inhibitor. Total RNA was isolated and subjected to RT-PCR to measure BAFF mRNA levels. *Upper*, BAFF mRNA expression normalized with that of β-actin is presented as the percentage change ± standard deviation of three separate experiments against the Pam3CSK4 treatment group, set as 100% by densitometric analysis. *Lower*, a representative BAFF mRNA expression determined by RT-PCR. Asterisk (*) indicates statistical significance at *p* < 0.05 between the Pam3CSK4 and other treatment groups.

**Figure 8:**
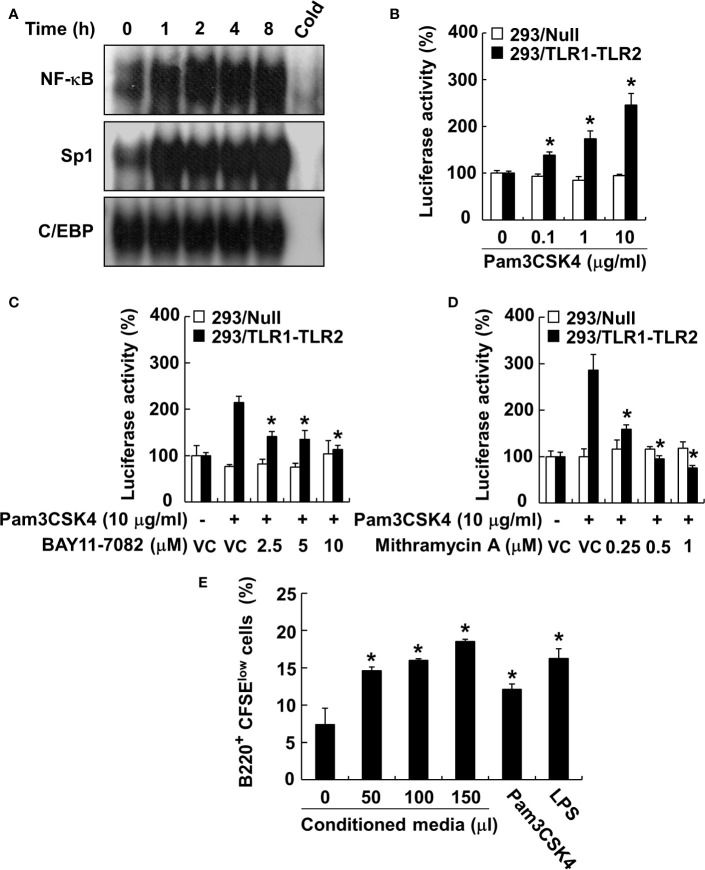
NF-κB and Sp1 are major transcription factors involved in the Pam3CSK4-induced BAFF expression. **(A)** Nuclear extracts were prepared from DCs stimulated with 10 μg/ml of Pam3CSK4 for 0–8 h and incubated with ^32^P-labeled oligonucleotides containing NF-κB, Sp1, or C/EBP consensus sequences. One picomole of an unlabeled probe (Cold) was used in a competition assay to confirm specific binding. Reaction products were separated on a polyacrylamide gel and visualized by autoradiography. **(B)** The 293/TLR1-TLR2 or 293/Null cells transiently transfected with the mouse BAFF promoter luciferase reporter construct together with pRL-TK vector were stimulated with 10 μg/ml of Pam3CSK4 for 24 h. **(C, D)** The cells were pre-treated with indicated inhibitor (BAY11-7082 for NF-κB inhibition or mithramycin A for Sp1 inhibition) for 1 h followed by stimulation with 10 μg/ml of Pam3CSK4 for an additional 24 h. DMSO (0.1%) was used as a vehicle control (VC) for each inhibitor. After the stimulation, cytosolic extracts were subjected to the luciferase activity assay. Values are the mean ± standard deviation of triplicates. Asterisk (*) indicates a significant difference at *p* < 0.05 between the non-treatment and treatment groups **(B)**, and between the Pam3CSK4 and other treatment groups **(C, D)** within the same cell-line, respectively. **(E)** The CFSE-labeled splenocytes were stimulated with the indicated amount of conditioned media of DC stimulated with Pam3CSK4, 10 µg/ml of Pam3CSK4, or 1 µg/ml of LPS for 72 h. B-cell proliferation was then determined by monitoring CFSE levels of the B220-positive cell population using flow cytometry. Values are the mean ± standard deviation of triplicates. Asterisk (*) indicates a significant difference at *p* < 0.05 between the non-treatment and treatment groups.

Because the MyD88-dependent pathway promotes phosphorylation of MAP kinases, subsequently leading to the activation of transcription factors, such as NF-κB (26), we examined the phosphorylation of MAP kinases by Pam3CSK4 in DCs. The results showed that Pam3CSK4 induced the phosphorylation of all MAP kinases tested (**Supplementary Figures 1A–D**). At the same time, we also optimized the concentration of MAP kinase–specific inhibitors (SP600125 and JNK V inhibitor for JNK inhibition, SB202190 for p38 inhibition, or PD98059 for ERK inhibition) to effectively suppress the Pam3CSK4-induced MAP kinase phosphorylation for further experiments (**Supplementary Figures 1A–D**). Under the optimized condition, we investigated the role of MAP kinases in Pam3CSK4-induced BAFF expression in DCs using MAP kinase–specific inhibitors. Among them, Pam3CSK4-induced BAFF mRNA expression was suppressed only by JNK MAP kinase-specific inhibitors, SP600125 and JNK V inhibitor, in a dose-dependent manner (**Figures 7A–D**). To determine which transcription factors are important for the transcriptional regulation of BAFF expression by Pam3CSK4, we examined the DNA-binding activities of NF-κB, C/EBP, and Sp1 transcription factors. C/EBP together with NF-κB is considered a crucial transcription factor needed for the expression of cytokines belonging to the TNF superfamily (26, 46), and Sp1 interacts synergistically with NF-κB to generate maximal activation of NF-κB (47). As shown in **Figure 8A**, NF-κB and Sp1 DNA-binding activity, but not that of C/EBP, was increased by Pam3CSK4 stimulation. These results imply that NF-κB and Sp1 transcription factors might be essential for the expression of BAFF by Pam3CSK4. To examine whether Pam3CSK4 can control BAFF expression at the transcriptional level, we transiently transfected 293/TLR1-TLR2 cells with the mouse BAFF promoter luciferase reporter construct and measured BAFF promoter activity after Pam3CSK4 stimulation. As shown in **Figure 8B**, BAFF promoter activity increased dose-dependently following Pam3CSK4 stimulation. Furthermore, we examined the role of NF-κB and Sp1 in transcriptional regulation of the Pam3CSK4-induced BAFF expression using specific inhibitors, BAY11-7082 for NF-κB inhibition and mithramycin A for Sp1 inhibition. As shown in **Figures 8C**, **D**, Pam3CSK4-induced BAFF promoter activity was suppressed by BAY11-7082 or mithramycin A in a dose-dependent manner, suggesting that NF-κB and Sp1 are essential transcription factors for transcriptional regulation of the Pam3CSK4-induced BAFF expression in DCs. Next, to determine whether BAFF from the Pam3CSK4-stimulated DCs can induce B-cell proliferation, splenocytes were stimulated with various amounts of the conditioned media from the Pam3CSK4-stimulated DCs. As Pam3CSK4 or LPS, the conditioned media enhanced splenic B-cell proliferation in a dose-dependent manner, providing an evidence that bacterial lipoproteins can indirectly promote B-cell proliferation (**Figure 8E**).

## Discussion

Microbial products can directly promote B-cell proliferation and activation by interacting with B cells and indirectly by modulating cytokine production of immune cells, such as IL-4, IL-5, TGF-β, or BAFF (2). Although direct B-cell activation by bacterial lipoproteins has been reported (27, 28), the mechanisms for indirect B-cell activation are poorly understood. In this study, we found that bacterial lipoproteins possess a BAFF-inducing ability in DCs. This finding is supported by relatively lower BAFF expression levels of a lipoprotein-deficient *S. aureus* (*Δlgt)* strain compared with that of its wild-type strain. In addition, Pam3CSK4 induced the highest level of BAFF expression among the tested microbial products, including LTA, poly(I:C), PGN, and flagellin. Pam3CSK4-induced BAFF expression was mediated by MyD88-dependent signaling pathways, followed by activation of JNK MAP kinase, NF-κB, and Sp1, leading to an increase in BAFF transcription. In fact, BAFF from the Pam3CSK4-stimulated DCs enhanced B-cell proliferation. These results demonstrate that bacterial lipoproteins are major microbial products inducing BAFF expression in DCs suggesting a possible mechanism for indirect B-cell activation.

However, the BAFF-inducing ability of bacterial lipoproteins appears to vary by cell type. Pam3CSK4 has been reported to induce BAFF mRNA expression in mouse splenic DCs (48). In contrast, although poly(I:C) and LPS induced BAFF expression in human dermal fibroblasts (49) and basophils (50), respectively, Pam3CSK4 did not affect BAFF production in these cell types (49, 50). Previous studies also reported cell-type-dependent BAFF expression by microbial products. Both BAFF mRNA and protein levels increased in the presence of double-strand RNA in human airway epithelial cell-lines, but not in the presence of PGN, LPS, flagellin, or CpG ODN (51). In addition, BAFF production was induced by LPS, but not poly(I:C) or zymosan in mouse mesenchymal stem cells (52). As we found in the current study, the differences in BAFF-expression patterns by microbial products according to cell type are likely a result of the expression of different TLRs in each cell type. Another study similarly reported that salivary gland epithelial cells induced BAFF expression in response to TLR3 ligand, poly(I:C), but not to other TLR ligands, including R837, PGN, and CpG ODN, because these cells showed relatively higher TLR3 expression compared with other TLRs (53). However, differences in BAFF-expression patterns according to microbial products in DCs imply that each TLR ligand functions differently in B-cell activation and in distinct and indirect ways. For example, our current study found that bacterial lipoproteins can indirectly promote expansion and activation of B cells by stimulating DCs to induce BAFF while our previous study demonstrated that LTA indirectly suppresses LPS-induced B-cell proliferation through other cell types, including macrophages and DCs (27).

In the current study, we demonstrated that BAFF production by bacterial lipoproteins was mediated through TLR2. In fact, BAFF expression by Pam3CSK4 was eliminated in DCs derived from TLR2-deficient mice and BAFF promoter activity was dose-dependently enhanced by Pam3CSK4 *via* TLR2/1. However, other tested TLR2 ligands, such as LTA and zymosan, did not affect BAFF expression in DCs. These observations can be explained by two potential mechanisms related to the inhibitory effects of these TLR2 ligands on their TLR2 activation. First, TLR2 ligands may utilize TLR co-receptors to weaken TLR2 activation. Although CD36 is needed for TLR2 activation leading to NF-κB, CD36 can also be involved in NF-κB inactivation by activating peroxisome proliferator-activated receptor-γ (PPAR-γ) (54). For example, because LTA binding to CD36 (55) and its PPAR-γ activation (56) has been reported, LTA may use the CD36/PPAR-γ signaling axis to suppress TLR2, resulting in NF-κB inactivation. Second, TLR2 ligands may induce and utilize the negative regulator of the TLR signaling pathway. In our previous study, LTA from *Enterococcus faecalis* diminished IL-8 induced by LPS from *Aggregatibacter actinomycetemcomitans* or *Porphyromonas gingivalis* in human periodontal ligament cells *via* the IRAK-M pathway, which is a negative regulator of the TLR signaling pathway (57, 58). These TLR2 inhibitory mechanisms seem to differ depending on properties of each TLR2 ligand. Since CD36 co-receptor binds with negatively charged moieties of its ligand more avidly (59), LTA, a negatively-charged cell wall component of Gram-positive bacteria, might easily bind with CD36 and induce NF-κB inactivation than Pam3CSK4 having positive charge. For suppression of TLR signaling by IRAK-M, different intracellular signaling cascades by TLR2 homodimer and heterodimer might determine the IRAK-M induction. TLR 2/1 and TLR2/6 heterodimer recruited TIRAP/MyD88 and TIRAP/TIRAP as adaptor molecules for their down-stream signaling pathway, respectively (60), whereas adaptor molecule recruitment by TLR2 homodimer, need for sensing of LTA, was still not fully understood. Moreover, since LTA was weaker than Pam2CSK4 in TLR2-activating capacity as demonstrated by our previous study using NF-κB reporter cell-line, CHO/CD14/TLR2 (61), we proposed that TLR2 activation by LTA *via* TLR2 homodimer is likely to be weaker than that of lipopeptides through TLR2 heterodimer, which is limited to induce IRAK-M, but not BAFF production.

Based on our study of TLR2- or MyD88-deficient mice and the DNA-binding activity of NF-κB, BAFF induction by Pam3CSK4 appears to be mediated by a TLR2/MyD88/NF-κB activation. Concomitantly, previous studies showed that NF-κB is a key transcriptional factor mediating LPS-induced BAFF expression in macrophages (62, 63), demonstrating that BAFF induction by bacterial products is commonly mediated by an MyD88-dependent pathway involving NF-κB activation *via* degradation of an IκB kinase complex (26). However, the role of JNK MAP kinase in BAFF production remains unclear. Previous studies revealed that JNK MAP kinase upregulated Sp1 activation for maximal activation of NF-κB and Sp1 stability (64, 65), and leading to BAFF mRNA induction (35), whereas other studies demonstrated that JNK MAP kinases are only involved in the secretion of BAFF by affecting furin-like convertase enzyme activity rather than BAFF gene expression (66, 67). However, the decreased levels of BAFF mRNA expression due to the JNK MAP kinase inhibitor and the enhanced Sp1 activity by Pam3CSK4 suggest that JNK MAP kinase is likely involved in BAFF mRNA expression *via* Sp1 activation rather than BAFF secretion.

In this study, we demonstrated that bacterial lipoproteins are major bacterial products with a strong BAFF-inducing capacity in DCs and also confirmed that indirect B-cell activating capacity of bacterial lipoproteins *via* BAFF production is beyond its direct effect. On the other hand, previous data from clinical and *in vivo* studies revealed increased expression levels of BAFF during bacterial infections by *Mycoplasma pneumoniae*, *Mycobacterium tuberculosis*, and *Pseudomonas aeruginosa* (68). In addition, increased levels of serum BAFF appear to correlate with disease severity in bacterial infectious diseases, such as tuberculosis (69). Since BAFF can promote inflammatory responses by inducing excessive Th1 and Th17 responses (16, 68), BAFF production by bacterial lipoprotein might be closely related with progression and persistence of the bacterial infectious diseases. Moreover, bacterial lipoproteins have a potency to produce cytokines having BAFF-inducing ability, such as TNF-α and IL-10 (70, 71), leading to synergistically enhanced BAFF production in DCs. Therefore, these observations propose a possible explanation for how bacterial lipoprotein might be involved in initiation and progression of inflammatory responses during bacterial infection.

## Data Availability Statement

All datasets presented in this study are included in the article/**Supplementary Material**.

## Ethics Statement

The animal study was reviewed and approved by the Seoul National University Institutional Animal Care and Use Committee (Approval Number: SNU-070221-2). Experiments were conducted in accordance with guidelines established by the Seoul National University Institutional Animal Care and Use Committee.

## Author Contributions

JI and SH designed research. JI, JB, DL, and DP carried out experiments. JI, DL, and SH analyzed and interpreted data. JI, DL, O-JP, C-HY, and SH prepared and reviewed the manuscript. All authors contributed to the article and approved the submitted version.

## Funding

This work was supported by grants from the National Research Foundation of Korea, which is funded by the Korean government (NRF-2018R1A5A2024418 and NRF-2019R1A2C2007041).

## Conflict of Interest

The authors declare that the research was conducted in the absence of any commercial or financial relationships that could be construed as a potential conflict of interest.
