# Supplementary material for: Bacterial Lipoproteins Induce BAFF Production via TLR2/MyD88/JNK Signaling Pathways in Dendritic Cells
Source: Front Immunol. 2020 Oct 2;11:564699. doi: 10.3389/fimmu.2020.564699 (PMC7566273; doi:10.3389/fimmu.2020.564699)
Supplement: Supplementary file 1 [file Presentation_1.pptx]

## Slide 1
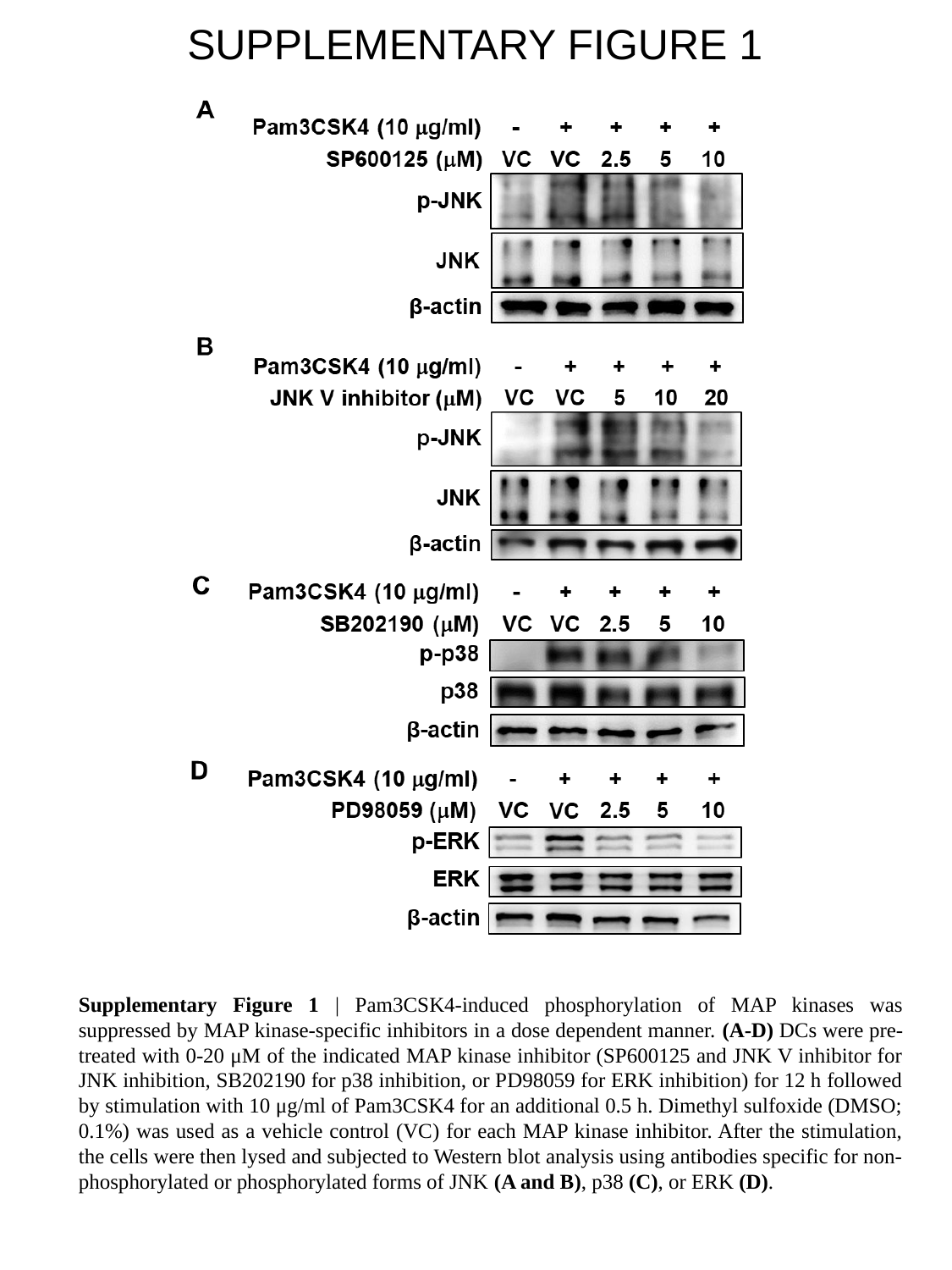

SUPPLEMENTARY FIGURE 1
Supplementary Figure 1 | Pam3CSK4-induced phosphorylation of MAP kinases was suppressed by MAP kinase-specific inhibitors in a dose dependent manner. (A-D) DCs were pre-treated with 0-20 μM of the indicated MAP kinase inhibitor (SP600125 and JNK V inhibitor for JNK inhibition, SB202190 for p38 inhibition, or PD98059 for ERK inhibition) for 12 h followed by stimulation with 10 μg/ml of Pam3CSK4 for an additional 0.5 h. Dimethyl sulfoxide (DMSO; 0.1%) was used as a vehicle control (VC) for each MAP kinase inhibitor. After the stimulation, the cells were then lysed and subjected to Western blot analysis using antibodies specific for non-phosphorylated or phosphorylated forms of JNK (A and B), p38 (C), or ERK (D).
